# Germination and Plantlet Regeneration of Encapsulated Microshoots of Aromatic Rice (*Oryza sativa* L. Cv. MRQ 74)

**DOI:** 10.1100/2012/578020

**Published:** 2012-08-02

**Authors:** Rosna Mat Taha, Azani Saleh, Noraini Mahmad, Nor Azlina Hasbullah, Sadegh Mohajer

**Affiliations:** ^1^Institute of Biological Sciences, Faculty of Science, University of Malaya, 50603 Kuala Lumpur, Malaysia; ^2^International Education Centre (INTEC), MARA University of Technology, Section 17 Campus, 40200 Shah Alam, Selangor, Malaysia; ^3^Department of Agricultural Sciences, Faculty of Technical and Vocational Education, Sultan Idris Education University, 35900 Tanjung Malim, Perak, Malaysia

## Abstract

Plant tissues such as somatic embryos, apical shoot tips, axillary shoot buds, embryogenic calli, and protocom-like bodies are potential micropropagules that have been considered for creating synthetic seeds. In the present study, 3–5 mm microshoots of *Oryza sativa* L. Cv. MRQ 74 were used as explant sources for obtaining synthetic seeds. Microshoots were induced from stem explants on Murashige and Skoog (MS) medium supplemented with 1.5 mg/L benzylaminopurine (BAP). They were encapsulated in 3% (w/v) sodium alginate, 3% sucrose, 0.1 mg/L BAP, and 0.1 mg/L **α**-Naphthalene acetic acid (NAA). Germination and plantlet regeneration of the encapsulated seeds were tested by culturing them on various germination media. The effect of storage period (15–30 days) was also investigated. The maximum germination and plantlet regeneration (100.0%) were recorded on MS media containing 3% sucrose and 0.8% agar with and without 0.1 mg/L BAP. However, a low germination rate (6.67%) was obtained using top soil as a sowing substrate. The germination rate of the encapsulated microshoots decreased from 93.33% to 3.33% after 30 days of storage at 4°C in the dark. Therefore, further research is being done to improve the germination rate of the synthetic seeds.

## 1. Introduction

Rice is the most important food crop for a large proportion of the word's human population especially in East, South, Southeast Asia, the Middle East, Latin America, and the West Indies. In 2008, international rice price rose greatly due to general upward trend in grain prices caused by droughts, increased use of grains animal feed, and so forth, has led to worldwide food crisis. This caused the domestic rice price in Malaysia increase almost double. The only way to protect and stabilise local price is to increase local rice production. Hence, synthetic seed technology could be an alternative and potential tool for the propagation of rice. 

Nowadays, the encapsulation technique for producing synthetic seeds has become an important asset in micropropagation. Encapsulation of somatic embryos, apical and axillary shoot buds, and regeneration of whole plants from them have been reported for a number of plant species [1–4]. The use of unipolar axillary shoot buds and apical shoot tips in creating the synthetic seeds have been reported in many plants species such as *Actinidia deliciosa*, *Brassica campestris*, *Malus pumila *Mill, *Zingerber officinale* Rose, and *Syringa vulgaris* L. [5]. Axillary shoot buds and apical shoot tips are suitable for encapsulation studies of artificial seeds as they possess great potential for plant development from pre-existing meristematic tissue. In addition, the use of axillary shoot buds and apical shoot tips would also ensure a genetic uniformity and stability in the regenerants. However, information about production of artificial seeds from apical shoot tips or microshoots in rice is extremely limited. Therefore, in the present study, different encapsulation matrix and efficacy in the plantlet regeneration of encapsulated microshoots of *Oryza sativa* L. Cv. MRQ 74 were investigated. Comparison on morphological structure such as stomata density between intact plant, *in vitro* plantlet, and plantlet from synthetic seed were also carried out.

## 2. Materials and Methods

### 2.1. Explant Source

Microshoots of *Oryza sativa* L. Cv. MRQ 74 were induced from stem explants. Prior to this, dehusked mature seeds of rice were surface sterilized by soaking and shaking in 70% (v/v) clorox with two drops of 1 mL/L tween 20 followed by 50%, 30%, 20%, and 10% (v/v) clorox. Shaking the material during sterilization would obviously enhance the effectiveness of the process. Each treatment lasted approximately one minute. The dehusked seeds were then rinsed once in sterilised distilled water. Finally, the seeds were rinsed in 70% (v/v) ethanol for one minute, followed by three times in sterilised distilled water for complete removal of clorox and ethanol in lamina flow. 

The sterilised seeds were then cultured onto MS medium [6] containing sucrose (30 g/L) and agar (8 g/L). Ten seeds were placed per culture tube. The cultures were incubated in the culture room at 25 ± 1°C under 16 hours light and 8 hours dark with 1000 lux of light intensity. The seeds started to germinate after 3 to 4 days to form plantlets. The plantlets were maintained in the culture room for 5 weeks before they were used as the explants in obtaining microshoots for synthetic seeds production.

The stem explants were approximately cut into 5.0–10.0 mm segments and cultured onto MS medium supplemented with 3.0% (w/v) sucrose and 0.8% (w/v) technical agar fortified with 1.5 mg/L BAP for microshoots induction. The pH of the medium employed in this experiment was adjusted to 5.8 prior to autoclaving process at 121 °C, 105 kPa for 21 minutes. The cultures were kept in the culture room at 25 ± 1°C under 16 hours light and 8 hours dark with 1000 lux of light intensity. Microshoots (approx., 5.0 mm in length) were excised from cultures after 2 weeks in culture. The microshoots were carefully isolated and encapsulated.

### 2.2. Capsule Matrix and Encapsulation of Microshoots

Microshoots of 3–5 mm in length were used as explants source in obtaining synthetic seeds. Different encapsulation matrices were evaluated: (1) Ca-free MS (Duchefa) + distilled water, (2) Ca-Free MS + 3% sucrose, and (3) Ca-free MS + 3% Sucrose + 0.1 mg/L BAP (Sigma) + 0.1 mg/L NAA (Sigma). Microshoots were mixed in the encapsulation matrix consisted of 3% (w/v) sodium alginate (Sigma), added with MS basal liquid medium with or without 3% sucrose and plant growth hormone. For complexation, 0.2 M of calcium chloride solution (CaCl_2_·2H_2_O) was prepared in distilled water. The gel matrix and the complexing agent were autoclaved after adjusting the pH to 5.8. The microshoots were drawn up with some encapsulation matrix and dropped into CaCl_2_·2H_2_O solution using sterilized micro pipette. The encapsulated microshoots were left for 30 minutes for hardening. The beads containing one microshoot each were washed in sterilized distilled water to avoid sticking together and were retrieved using nylon mesh.

### 2.3. Germination Medium/Substrate

The beads were germinated on various germination media and substrates: (1) MS basal medium + 3% sucrose + 0.8% agar (MSO), (2) MS + 3% sucrose + 0.8% agar + 0.1 mg/L BAP, (3) MS + 0.8% agar, (4) tap water + 0.8% agar, (5) top soil, (6) top soil + tap water, and (7) top soil + 1/2 strength MS + 3% sucrose. All the culture media and substrates were autoclaved at 121 kPa for 21 minutes prior to be used. In testing the best encapsulation matrix, the nonencapsulated microshoots were used as control. The encapsulated and nonencapsulated microshoots were cultured onto MS basal medium. Meanwhile in determining the best culture medium and cultured substrate, MS basal medium with 3% sucrose and 0.8% agar was used as control using Ca-free MS + 3% sucrose + 3% sodium alginate + 0.1 mg/L BAP + 0.1 mg/L NAA as encapsulation matrix. Thirty replicates were used for each treatment. All the cultures were kept in the culture room at 25 ± 1°C, 16 hours light and 8 hours dark. The germination rate of the synthetic seeds and plantlets survival rate were recorded after 10 days and 30 days of culture, respectively.

### 2.4. Storage Period

The beads were also cold-stored in the incubator at 4°C prior to germination process. Thirty seeds were sown in MS basal medium for every 15 days interval. The germination rates were recorded after 4 weeks of sowing.

### 2.5. Microscopic Studies (Scanning Electron Microscopy-SEM)

Scanning electron microscope (SEM) was used to observe the differences between *in vivo* (intact) and *in vitro* leaf. Observations and comparisons were made on the differences of number of stomata and trichomes. Standard methods and procedures for the preparation of samples for SEM process as described by Islam et al. [7] were followed.

## 3. Statistical Analysis

All the experiments were repeated trice and thirty replicates were used. The effect of different treatments was quantified as mean ± SE and the data were subjected to statistical analysis using Duncan's multiple range test (DMRT) at 5% level significance [8].

## 4. Results and Discussion

### 4.1. Capsule Matrix and Encapsulation of Microshoots

**Figure 1:**
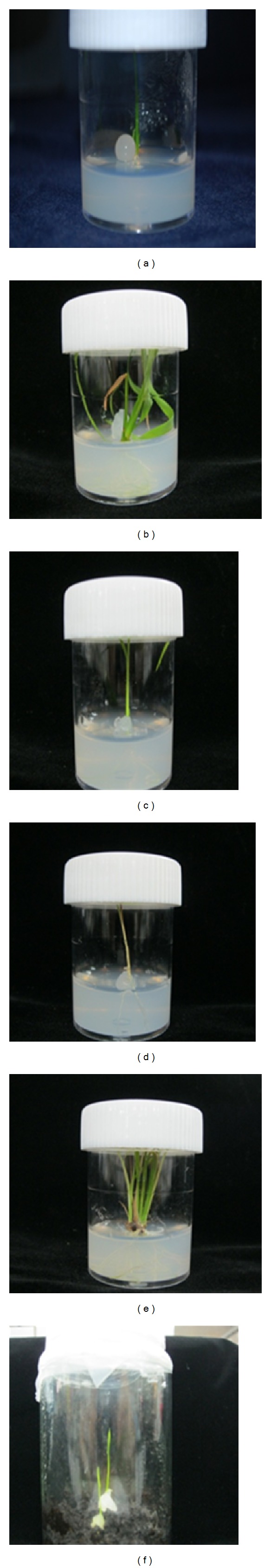
Synthetic seed germination on, (a) MS medium, two weeks old, (b) MS medium after one month, (c) MS medium + 0.1 mg/L BAP, (d) tap water + agar, (e) capsule matrix containing 0.1 mg/L BAP + 0.1 mg/L NAA, (f) top soil + tap water.

In the present study, the encapsulated and nonencapsulated microshoots showed 100% germination rate and had a high potential to be converted into plantlets on cultured medium. The highest survival rate which was 100% found using Ca-free MS + 3% sucrose, while nonencapsulated microshoots (control) showed the lowest survival rate of plantlet (90.0%) (Table 1). The survival rate of the plantlets significantly reduced when the encapsulation matrix only contain 3% sodium alginate with the addition of MS basal (free calcium) and distilled water. It is interesting to note that the addition of 0.1 mg/L BAP and 0.1 mg/L NAA did not influence the germination rate of the synthetic seeds (Figure 1(e)).

In the previous experiment, the optimum regeneration medium of rice (*Oryza sativa* L. Cv. MRQ 74) was MS medium supplemented with 0.1 mg/L BAP + 0.1 mg/L NAA (Data not shown). Therefore, the same types and concentrations of hormones were added in capsule matrix to stimulate the emergence of shoot and root of encapsulated microshoots. However, the use of these hormones had no significant effect on synthetic seeds germination. All treatments with and without hormone showed 100% germination rate. In fact, plantlets survival rate decreased to 96.67% compared to capsule matrix containing MS medium without growth hormone. For production of synthetic seeds from apical shoot tips and axillary shoot buds, these organs are usually first treated with auxins for root induction. However, Bapat and Rao [9] reported that mulberry plantlets were obtained from alginate encapsulated shoot buds without any specific root induction treatment. The same finding was reported on banana [3]. Roy and Mandal [10] also stated that embryos and pro-embryos of elite indica rice (*Oryza sativa* L. Var IR 72) developed into plantlets on MS basal medium without any phytohormones. These results were in line with the findings of the present study.

Synthetic seeds germination was affected by sucrose concentration in the capsule matrix.

Synthetic seeds with no sucrose in the capsule matrix had significantly lower plantlets survival rate as compared with synthetic seeds with sucrose in encapsulation matrix. It has been previously reported that the inoculated somatic embryos with various concentrations of sucrose (0, 30 and 60 mg/L) gave synthetic seeds germination to 43, 57, and 46% respectively [11]. Other studies have shown that low germination and conversion capacity of synthetic seeds is due to absence of nutritive tissues like the endosperm of the natural seed [12]. These results indicated that coating material and the concentration of the coating material are important limiting factors for the synthetic seed technology.

### 4.2. Germination Medium/Substrate

The highest germination rate (100%) was recorded on MS basal medium (MSO), MS medium supplemented with 0.1 mg/L BAP, tap water + 0.8% agar, top soil + tap water, and top soil + 1/2 strength MS + 3% sucrose (Table 2). Top soil showed the least preferred germination substrate with 6.67% germination rate. However, the survival rate of plantlets varied from 100% to 0.0%. The maximum survival rate (100%) was observed on MS media with (Figure 1(c)) and without 0.1 mg/L BAP (Figures 1(a) and 1(b)) followed by tap water + 0.8% agar (93.33%, Figure 1(d)), MS + 0.8% agar (90.0%), top soil + tap water (36.67%, Figure 1(f)), top soil + 1/2 strength MS + 3% sucrose (30.0%), and top soil (0.0%).

Sucrose is not only an important substance in capsule matrix but also in culture media. The presence of sucrose in germination medium showed significant effect on germination rate and plantlets survival rate of *Oryza sativa* L. Cv. MRQ 74. Similar findings were reported on other species such as *Psidium guajava* L. With increasing concentration of sucrose (3–9%) in medium, the percentage of germination of encapsulated somatic embryos of *Psidium guajava* L. decreased significantly [13]. Taha et al. [14] found that MS without hormones supplemented with 30 g/L sucrose was the best substrate for germination of the synthetic seeds of *Saintpaulia ionantha* Wendl. The use of tap water in culture medium and substrate (top soil) gave maximum germination rate too (100%). However, plantlets survival rates dropped to 36.67%, 30.00%, and 0.0% in top soil + tap water, top soil + 1/2 strength MS + 3% sucrose, and top soil only, respectively, after 30 days. The most abundant minerals dissolved in water are salts of calcium, magnesium, ferrous iron, and manganese. Among the macronutrients required for plant cell and tissue growth are calcium and magnesium. In this study, plantlet survival rate is significantly higher in top soil + tap water (36.67%) compared to top soil + 1/2 strength MS + 3% sucrose (30.00%). However, we did not determine the mineral content of tap water used in this study. The mineral content of tap water varies considerably from place to place. For example, mineral levels of tap water vary among North American cities and even among different water sources within the same city [15]. The mineral content of water reflects the nature of the geologic formation with which the water has been in contact. 

### 4.3. Storage Period

The encapsulated microshoots with MS medium supplemented with 0.1 mg/L BAP + 0.1 mg/L NAA, 3% sucrose, and 0.8% agar gave 93.33% germination without storage (Table 3). The viability of seeds had fallen from 93.33% to 3.33% after one month storage at 4°C. Storage conditions such as temperature and period of storage are important factors to determine the regeneration frequency of the stored encapsulated propagules. Storage of synthetic seeds using an alginate encapsulation protocol has been attempted in a few species, with minimal success [1, 16, 17]. The synthetic seeds of sweet corn were germinated to 43 and 55% after 2 weeks of storage under 15 ± 2°C and 25 ± 2°C, respectively [11]. In the present study, low germination rate (16.67%) was recorded after 2 weeks of storage at 4°C. Therefore, further research for the development of a better technique is required. For example, increasing the storage temperature may increase regeneration levels. Elvax 4260 (ethylene vinyl acetate acrylic acid terpolymer, Du Pont, USA) could be used for coating the capsules to avoid rapid water loss when calcium alginate capsules are exposed to the ambient atmosphere [5]. 

### 4.4. Microscopic Studies (Scanning Electron Microscopy-SEM)

**Figure 2:**
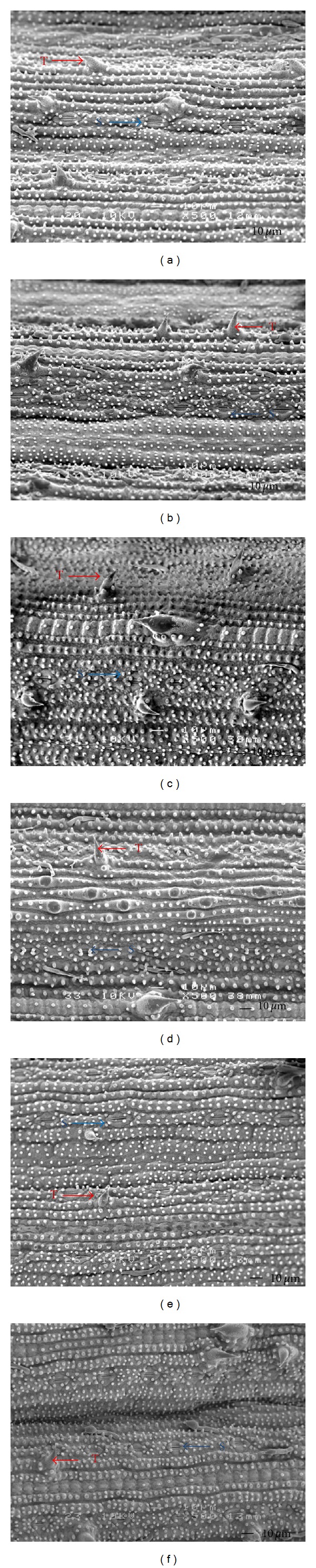
Scanning electron micrograph showing adaxial (a) and abaxial (b) surfaces of *in vitro* leaf of plantlet from synthetic seed of *Oryza sativa* L. Cv. MRQ 74, adaxial (c) and abaxial (d) surfaces of leaf from *in vivo* (intact) plant, adaxial (e), and abaxial (f) surfaces of *in vitro* leaf of plantlet from culture medium containing 0.1 mg/L BAP and 0.1 mg/L NAA. S: stomata, T: trichomes.

The microscopic studies of the structure of *in vivo* (intact) and *in vitro* leaves showed that the stomata apparatuses were generally triangular in shape. The number of stomata was higher on abaxial surfaces of *in vivo* and *in vitro* (plantlet from synthetic seed) leaves. However, a comparable number of stomata were observed on abaxial and adaxial surfaces of leaf from 0.1 mg/L BAP + 0.1 mg/L NAA treatment. More trichomes were seen on adaxial surface of intact leaf compared with *in vitro* leaf, grown on MS medium supplemented with 0.1 mg/L BAP + 0.1 mg/L NAA (Figure 2).

## 5. Conclusions

It can be concluded that this study was successful in determining the most suitable capsule matrix (Ca-free MS + 3% sucrose) and the optimum culture medium (MS medium + 3% sucrose with and without 0.1 mg/L BAP) for the maximum germination rate (100%) and plantlets survival rate (100%) of *Oryza sativa* Cv. MRQ 74. The encapsulated and nonencapsulated microshoots developed into plantlets on MS basal medium without any growth hormones. Based on scanning electron microscopic (SEM) studies, leaves derived from stem explants cultured on MS medium supplemented with 0.1 mg/L BAP + 0.1 mg/L NAA showed higher stomata number on both surfaces. However, there was no significant differences between *in vivo* and *in vitro* in terms of stomata number on both adaxial and abaxial leaf surfaces, indicating that no morphological changes had occurred. In order to confirm these findings, further histological studies on leaf tissues grown *in vivo* and *in vitro* should be conducted.

## Figures and Tables

**Table 1:** Growth response of encapsulated microshoots of *Oryza sativa* L. Cv. MRQ 74 in different capsule matrix after being transplanted onto MS media for 10 and 30 days.

Capsule matrix	Germination rate (after 10 days) (% ± SE)	Plantlets survival rate (after 30 days) (% ± SE)
Control	100.00 ± 0.00_a_	90.00 ± 0.55_a_
Ca-free MS + distilled water	100.00 ± 0.00_a_	93.33 ± 0.48_b_
Ca-free MS + 3% sucrose	100.00 ± 0.00_a_	100.00 ± 0.00_d_
Ca-free MS + 3% sucrose + 0.1 mg/L BAP + 0.1 mg/L NAA	100.00 ± 0.00_a_	96.67 ± 0.48_c_

Mean ± SE, *n* = 30. Mean with the same letter in the columns are not significantly different at *P* = 0.05.

**Table 2:** Effect of different sowing media/substrates on germination rate of synthetic seeds of *Oryza sativa* L. Cv. MRQ 74.

Sowing medium/substrate	Germination rate (after 10 days) (% ± SE)	Plantlets survival rate (after 30 days) (% ± SE)
MS basal medium + 3% sucrose + 0.8% agar (control)	100.00 ± 0.00_c_	100.00 ± 0.00_f_
MS + 3% sucrose + 0.8% agar + 0.1 mg/L BAP	100.00 ± 0.00_c_	100.00 ± 0.00_f_
MS + 0.8% agar	96.67 ± 0.48_b_	90.00 ± 0.55_d_
Tap water + 0.8% agar	100.00 ± 0.00_c_	93.33 ± 0.48_e_
Top soil	6.67 ± 0.48_a_	0.00 ± 0.00_a_
Top soil + tap water	100.00 ± 0.00_c_	36.67 ± 0.36_c_
Top soil + 1/2 strength MS + 3% sucrose	100.00 ± 0.00_c_	30.00 ± 0.55_b_

Mean ± SE, *n* = 30. Mean with the same letter in columns are not significantly different at *P* = 0.05.

**Table 3:** Effect of storage period (day) at 4 ± 1^°^C on germination of synthetic seeds of *Oryza sativa* L. Cv. MRQ 74 on MS basal medium.

Storage period (day)	Number of synthetic seeds	Number germinated	Germination rate (% ± SE)
0	30	28	93.33 ± 0.48_d_
15	30	5	16.67 ± 0.42_c_
30	30	1	3.33 ± 0.36_b_
45	30	0	0.00 ± 0.00_a_

Mean ± SE, *n* = 30. Mean with the same letter in the column is not significantly different at *P* = 0.05.
